# The thoracic surgery patient’s journey through the hospital – a pilot project on resource consumption and potentials for sustainability

**DOI:** 10.1007/s00423-025-03782-w

**Published:** 2025-07-01

**Authors:** Lena Katharina Knüvener, Sebastian Kalverkamp, Jan Spillner, Julia Wallqvist, Wiam Khader, Sebastian Ziemann, Julia Schuler, Rose Nangah Mankaa, Marzia Traverso, Linda Grüßer

**Affiliations:** 1 Department of Thoracic Surgery, University Hospital RWTH Aachen, Aachen, Germany; 2 Department of Anaesthesiology, University Hospital RWTH Aachen, Aachen, Germany; 3 Institute of Sustainability in Civil Engineering, RWTH Aachen University, Aachen, Germany

**Keywords:** Climate change, Sustainability, Thoracic surgery, Resource consumption

## Abstract

**Purpose:**

Medical societies around the globe are searching for ways to decrease the environmental impacts of patient care. This pilot project aims to identify potentials for more sustainability in clinical routine by investigating the resource consumption of thoracic surgery patients.

**Methods:**

This single-centre, observational, prospective pilot project was conducted at the RWTH Aachen University Hospital, Germany, from May 2023 to August 2023. Five patients with planned video-assisted-thoracoscopic surgery for removal of (suspected) lung cancer were included and followed throughout their treatment at the hospital. We recorded resource consumption for their direct care and investigated the share of disposable and reusable products and the packaging of disposable products. Additionally, we conducted a PubMed literature search on available life cycle assessments of the utilised products and investigated manufacturers’ online information on sustainability aspects of their products.

**Results:**

An average of 1254 disposable (75%) and reusable (25%) products were used per patient throughout their hospital journey. Most disposable products’ packaging contained plastic. We identified 30 publications that reported life cycle assessments. Manufacturers provided information on sustainability aspects for 10% of the products utilised.

**Conclusions:**

In-hospital patient care is resource intensive. Disposable products outnumbered reusable products at every stage of the patient’s journey and were mostly packaged in materials containing plastic. For the majority of products, no information concerning their environmental impact was accessible hampering informed purchasing choices by clinicians. Further efforts are essential to make environmental data available, leverage circular-economy systems, and ultimately decrease the environmental impacts of the healthcare sector.

**Supplementary Information:**

The online version contains supplementary material available at 10.1007/s00423-025-03782-w.

## Introduction

Climate change is considered the greatest global health threat of the 21 st century [1]. On the one hand, the healthcare sector will be burdened by the impacts of climate change [1]. On the other hand, it contributes significantly to climate change itself. It is estimated that the healthcare sector accounts for 4.4–4.6% of the global net emissions [2, 3]. Around 70% of this contribution is attributed to healthcare supply chains [2]. To achieve the 1.5 – degree goal set in the Paris Agreement 2015, CO_2_ emissions must be halved by 2030 and reach net-zero by 2050 [4]. Urgent actions are required to decrease the healthcare sector´s carbon footprint [5].

Several studies have been conducted to explore potentials for sustainability in various medical areas. For some healthcare products and procedures, life cycle assessments (LCAs) are available [6, 7]. In addition, the environmental impacts of single-use versus reusable medical products have been investigated [8, 9]. In one study, researchers examined material flows in the intensive care unit (ICU) and identified five disposable products as “hotspots” due to their high frequency of use and significant environmental impact (including global warming potential, agricultural land occupation and water usage) [10]. However, to identify areas for improvement in daily clinical routine, investigating resource consumption on a patient’s level is essential. In a study to examine the carbon footprint of treating 10 patients with septic shock energy consumption for heating, ventilation and air conditioning contributed the most to CO_2_ equivalent emissions [11]. Apart from these analyses, the amount of resources patients use throughout their journey in the hospital and their environmental impact remains largely unknown.

This pilot project was conducted to track thoracic surgery patients from pre- to post-surgery, evaluate which resources are used during their hospital treatment and collect information on their environmental impacts. We hope to provide insights into resource consumption in clinical routines and thus aid in the identification of research questions that should be prioritised in future projects on environmental sustainability and the establishment of circular-economy principles in the healthcare sector.

## Patients and methods

### Project design

In this single-centre, observational, prospective, exploratory pilot project, we investigated the resource consumption of five patients, who were treated with video-assisted-thoracoscopic-surgery (VATS) for removal of lung tissue at the RWTH Aachen University Hospital. This was chosen as an index procedure, because it is a common procedure in thoracic surgery. The project was performed in accordance with the Declaration of Helsinki and ethical approval was obtained from the Independent Ethics Committee of the RWTH Aachen University Hospital (EK23-042) on 24 April 2023. It was registered at the Open Science Framework Registry (osf-registrations-7dwtr-v1).

### Setting and participants

The RWTH Aachen University Hospital is a tertiary hospital in Germany. Approximately 250 VATS are performed per year. All patients aged ≥ 18 years who attended the Department of Thoracic Surgery’s outpatient clinic from 26 April to 30 June 2023 were screened for eligibility. Patients were included if they were planned to undergo a VATS due to (suspected) lung cancer and were capable of giving consent. Exclusion criteria were language barrier, emergency surgery and a planned post-surgery ICU stay. Informed consent was obtained in written form from eligible patients.

### Data collection

Data collection took place from 9 May 2023 until 9 August 2023. Patients were accompanied from their first visit in the outpatient clinic to their examinations, the operating room (OR), their pre- and postoperative stay at the ward and their postoperative aftercare at the outpatient clinic. Except for the stay at the ward, trained study staff documented all resources used on paper case report forms in real time. At the ward, patients were visited once a day and patients, doctors and nurses were asked about the patient’s resource consumption on the given day.

We recorded only items in direct proximity to the patient specifically used for their treatment, e.g., the OR staff´s clothing. Clothing of staff at the ward were not recorded because these were used for the treatment of all patients at the ward that day. Resource consumption in departments such as administration, laboratory, pathology, or sterilisation was also not considered because processes were in place for many patients at the same time. In addition, we excluded furniture and facilities, such as the patient’s bed, examination couches, tables and chairs. Furthermore, our investigation does not include an analysis of electricity, warming and cooling, or water usage because this data could not be provided by the hospital administration and cannot be directly influenced by clinicians.

### Outcome measures

#### Resources

The primary outcome was the number of products used for one patient throughout their journey through the hospital. To enable a better understanding of the deployment of products during each stage, we divided the journey into 4 categories: (1) thoracic surgery outpatient clinic (pre- and postoperative), examinations (pre- and postoperative) and preoperative anaesthesia consultation at the anaesthesia clinic; (2) surgery; (3) anaesthesia and recovery room; (4) ward.

Similar products in different sizes were grouped, e.g. sterile gauze swabs, adhesive dressings and syringes. We categorised the products as disposable and reusable products. Disposable products included items that were used for just the respective patient and were then disposed. Reusable items included items that could be used for multiple patients. We reviewed all disposable items´ individual packaging. If a product was packaged in a combination of paper and plastic, it was categorised as plastic.

To identify the disposable products that were used most frequently in our setting, we also calculated the total number of products that were used for all five patients. We also classified reusable products as surgical instruments, medical devices, medical products, textiles and others, and disposable products as medical products, pharmaceutics, cleaning and disinfecting agents and others. Medical products included products mainly used in healthcare whereas we sorted products such as paper sheets, razors, and headphones in the category “others”.

#### Manufacturer information

We searched online for manufacturer information concerning the sustainability of each product used. Every item’s name was searched in combination with the keywords “environment”, “sustainable” and “sustainability” from April 2023 to November 2023. Because no comprehensive, independent LCAs were publicly accessible, we considered information on any sustainability aspect mentioned directly in connection with the product as available sustainability information. Additionally, we searched for general sustainability reports from the manufacturers. We defined a “sustainability report” as a report that disclosed data regarding environmental protection/sustainability of the last year and/or set sustainability targets for the future. The report had to be from 2020 or more recent.

#### Literature search

We conducted a literature search from a clinician’s perspective to gather information about the environmental impact of the products used for our patients. We searched the term “life cycle assessment” in the United States National Library of Medicine’s PubMed database on 7 November 2023 for titles relating to healthcare. We screened all abstracts to identify publications with LCAs of medical products or procedures and eventually included publications with LCAs of products and procedures that were similar to the ones used for our patients.

#### Food and transportation

We categorised every meal (breakfast, lunch, dinner) as meat/fish, vegetarian or vegan. For breakfast and dinner, we calculated 400 g and for lunch 550 g per meal. Patients were queried about their estimated food leftovers. Daily food waste was then calculated by dividing the five patients´ total food waste by the total days they spent at the ward.

Concerning transportation, every patient was interviewed about the distance travelled to the hospital and the mode of transportation.

### Statistical analysis

All analyses were conducted using Excel Spreadsheet in Microsoft^®^ Excel for Office365 version 2402 (Microsoft Corporation, Washington, U.S). Continuous variables are presented as mean (standard deviation) and/or median [IQR] and categorical variables are presented as number (% of total sample).

## Results

**Fig. 1:**
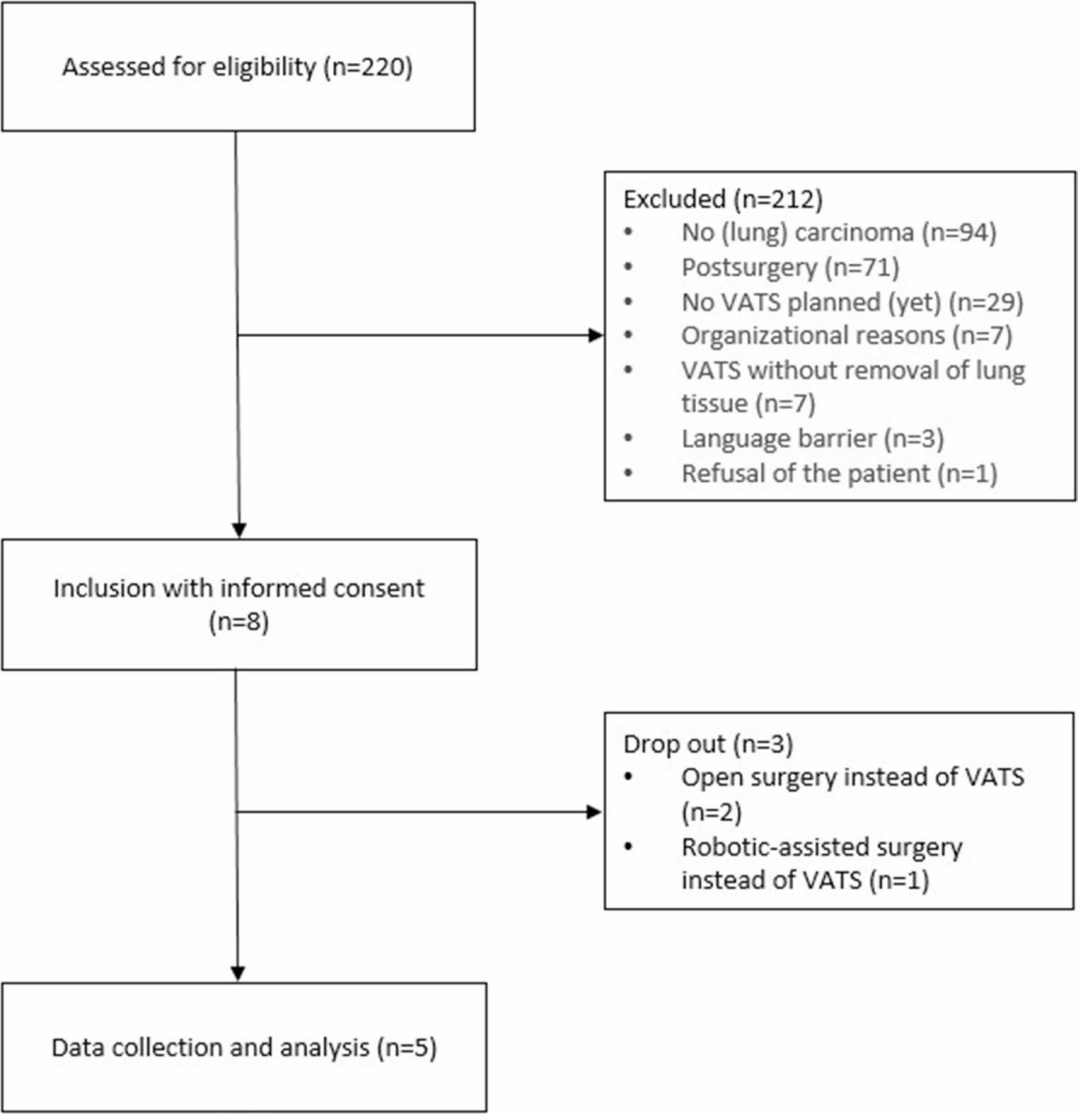
Overview of the screening and inclusion process

Overall, 220 patients visited the outpatient clinic during the project period and were screened for eligibility. The majority were excluded because they either did not have lung cancer or had already undergone VATS. Ultimately, five patients met the inclusion criteria and were enrolled in the project (Fig. 1). Their baseline characteristics are shown in Table 1.

**Table 1:** Patient baseline characteristics

	Sex (F/M)	BMI (kg/m^2^)	ASA	CCI	Surgery duration (min)	AN duration (min)	Days at ward	ICU stay (hours)
1	F	28.8	2	6	183	235	17	18
2	M	26.5	2	5	61	130	4	0
3	F	35.6	3	5	199	270	10	0
4	F	28.2	3	8	82	130	4	0
5	F	33	3	4	220	295	6	0
Median [IQR]	F: M 4:1	28.8 [28.2–33]	3 [2–3]	5 [5–8]	183 [82–199]	235 [130–270]	6 [4–10]	0 [0–18]

*Abbreviations*: *F *female, *M *male, *kg *kilogram, *m *meter, *BMI *body mass index, *ASA *American Society of Anesthesiologists classification, *CCI *Charlson-Comorbidity-Index, *min *minutes, *ANduration *duration of anaesthesia including patient positioning, *ICU *unplanned stay at intensive care unit

### Resources

**Fig. 2:**
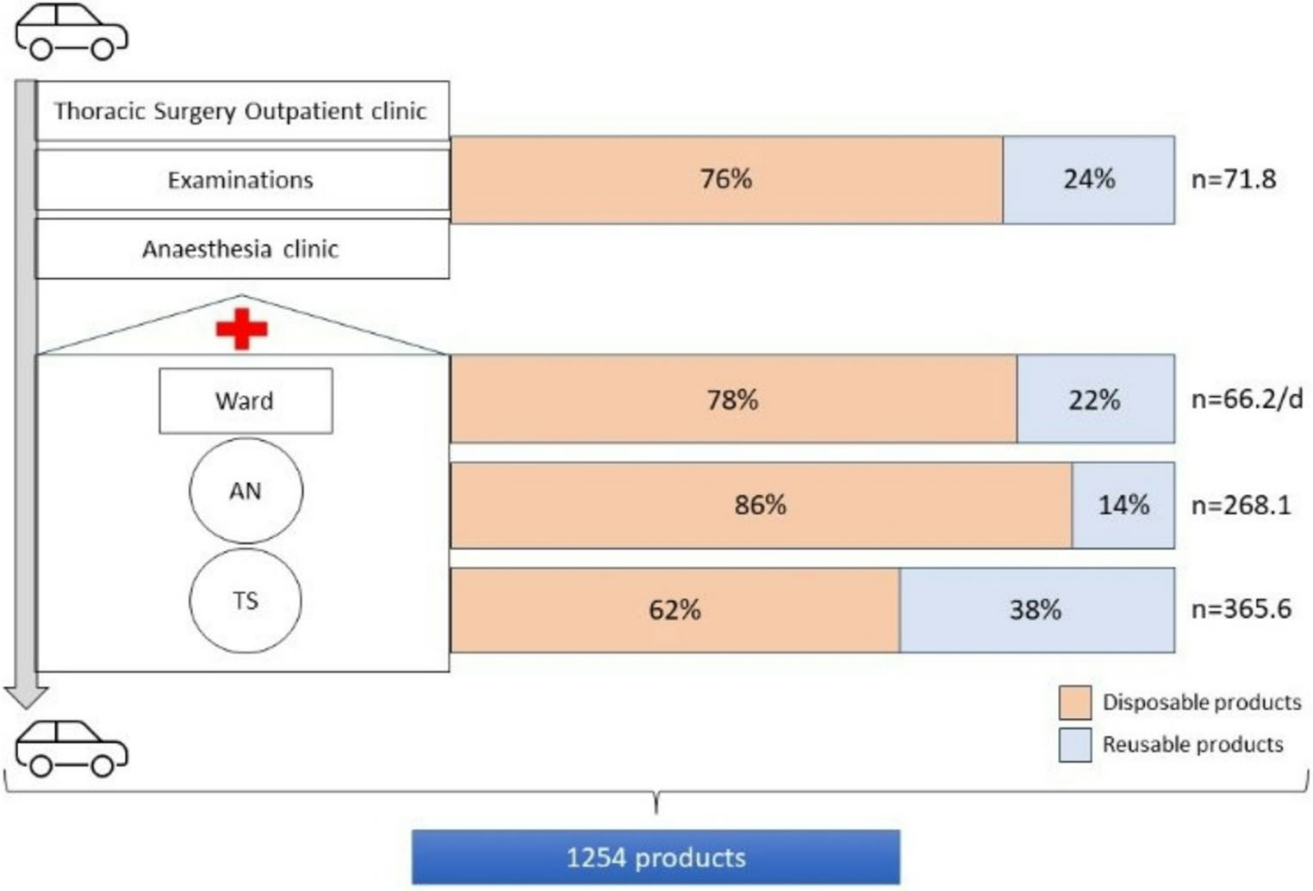
Consumption at the different stages in hospital careand the share of disposable and reusable items. Abbreviations: n – mean, AN – anaesthesia, TS – thoracic surgery, /d – per day

We found that for the treatment of one VATS patient a mean of 1254 (standard deviation 428) products were used. The majority of products were disposable (*n* = 946, 75%). Most products were utilised in the OR by anaesthesia and surgical staff (Fig. 2). At each stage of the hospital journey, the number of disposable products was higher than the number of reusable items. An overview of the patient’s product usage during the different stages can be found in Supplement 1.

**Fig. 3:**
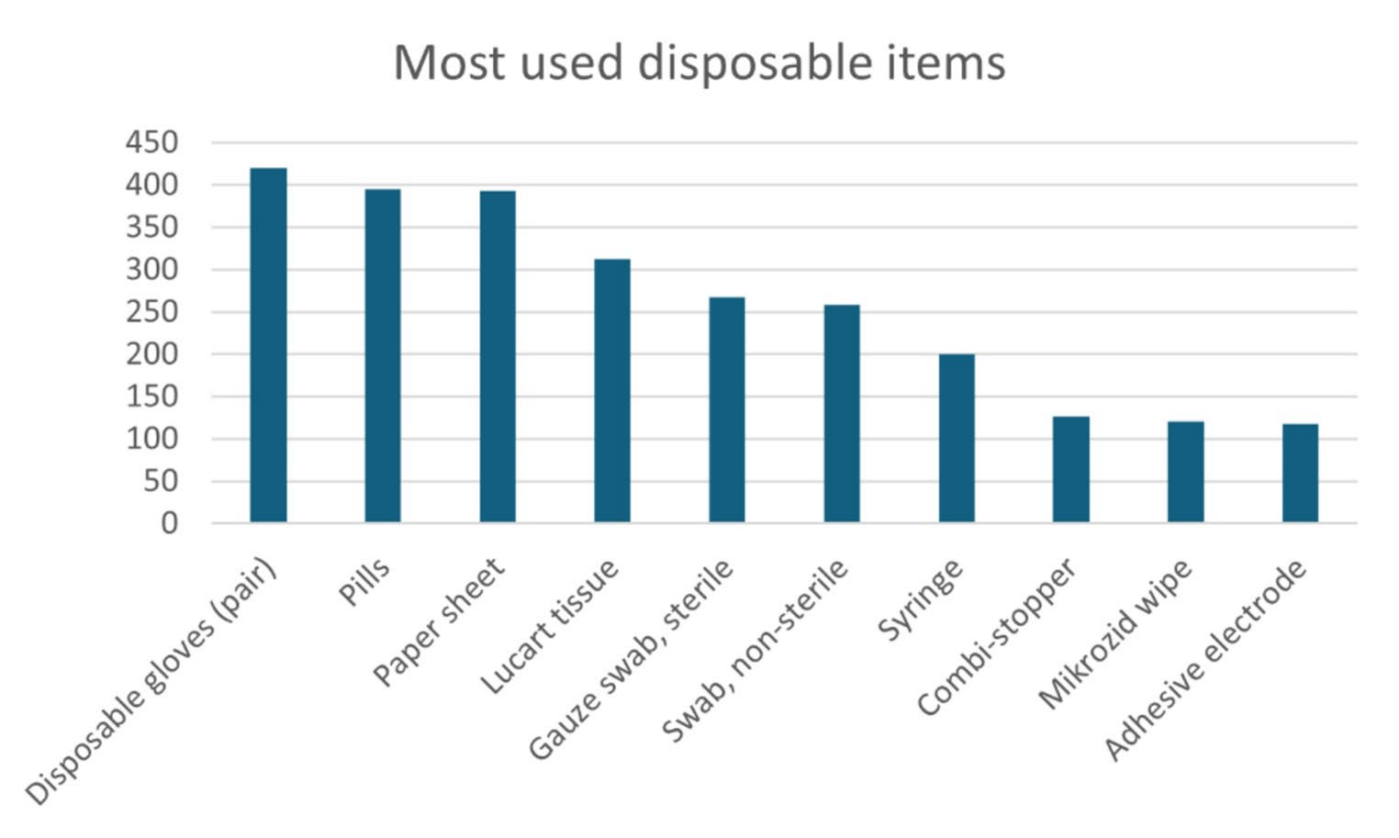
Most frequently used disposable products for five patients

For the treatment of all five patients 485 different products were applied one or multiple times. The categorization of the products and the disposable products´ packaging are presented in Table 2. Throughout all stages of the hospital stay gloves, pills and paper sheets were among the most frequently used disposable products (Fig. 3).

**Table 2:** Overview of reusable and disposable products and packing of disposable products

Total number of *different* products used for the treatment of five patients	485
**Reusable products,** ***n*** **(%)**	**224 (46.2%)**
- Surgical instruments	157 (70.1%)
- Medical devices	36 (16.1%)
- Medical products	15 (6.7%)
- Textiles	14 (6.3%)
- Others	2 (0.9%)
**Disposable products,** ***n*** **(%)**	**261 (53.8%)**
- Medical products	166 (63.6%)
- Pharmaceutics	77 (29.5%)
- Cleaning and disinfecting agents	11 (4.2%)
- Others	7 (2.7%)
Disposable products’ packaging	
> Without individual packaging	69 (26.4%)
> Individual packaging containing plastic	163 (62.5%)
> Individual packaging glass	23 (8.8%)
> Individual packaging paper	4 (1.5%)
> Individual packaging unknown	2 (0.8%)

### Manufacturer information

Out of the total of 485 different products used for the treatment of our five VATS patients, manufacturers provided information on sustainability aspects for 50 of them (10%). All products were provided by 156 manufacturers. About one third (37%) of them published a sustainability report in 2020 or later.

### Literature research

**Fig. 4:**
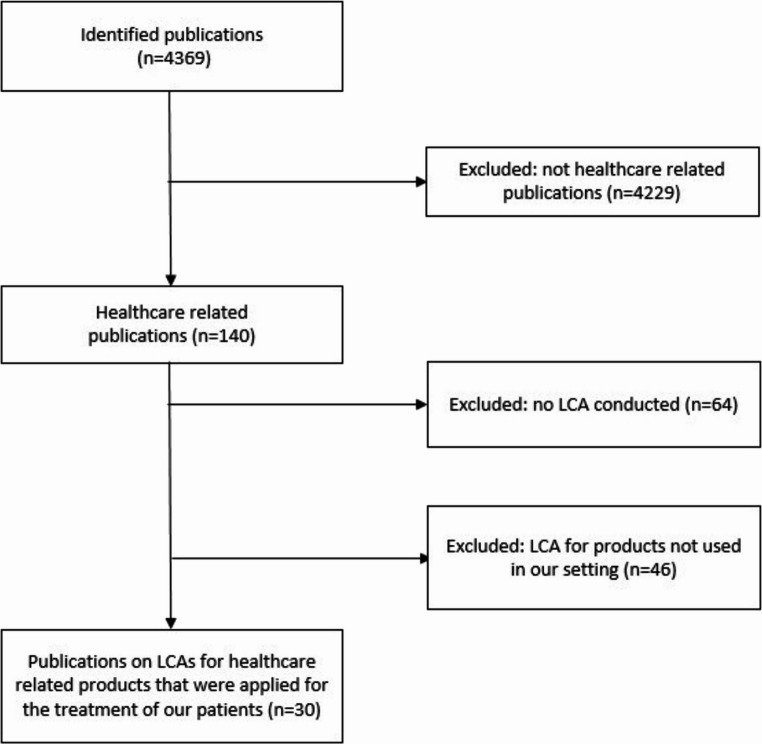
Overview of the PubMed literature research

Our literature search identified 4369 publications on LCAs (Fig. 4). Of these, 141 (3.2%) were healthcare-related. In total, 30 (0.7%) provided information on LCAs of similar products used in our setting. These publications primarily focused on personal protection equipment.

### Food and transportation

The five patients had 96 meals on 41 ward days (2.3 meals/patient/ward day). Of these, 70 (73%) meals contained meat or fish, 26 (27%) were vegetarian and none were vegan. The overall estimated food waste of five patients was 16.8 kg (39%) (410 gram/patient/ward day).

Patients travelled an average of 3.8 times to the hospital for their treatment (including preoperative examinations, inpatient hospital stay and one postoperative examination). In total, patients and/or their drivers travelled on average 190.8 km. All travelled the distances by car. Their cars’ emission classes according to the European emission standards were Euro 4 to 6.

## Discussion

In our pilot project, we found that a mean of 1254 products were utilised for the treatment of an average VATS patient at our hospital. Most products were applied in the OR. During all stages of the hospital stay, disposable products outnumbered reusable products. Most disposable products were packaged individually and the packaging most often contained plastic. For most products, neither did the manufacturer provide information on any sustainability aspects, nor could we find information on LCAs in the literature.

### Resources

In their material flow analysis of an ICU, Hunfeld et al. identified similar products such as disposable gloves and syringes as the most frequently used items for the treatment of their patients [10]. In their eco-audit, Grinberg et al. estimated that a cardiac surgery contributes 124.3 kg of CO_2_ equivalents with disposable medical products accounting for 89% of this total [12]. Indeed, single-use equipment has been identified as an emission hotspot in OR- and procedure-specific analyses [8]. There is now growing evidence that switching to reusable products can help reduce the environmental impact of surgical care [8, 9, 13]. Notably, streamlining and further developing cleaning, disinfection and sterilisation processes is essential to mitigate the environmental impact of reusable products. Yet, to date, little independent information about weighing the ecological pros and cons of disposable and reusable products is available to clinicians, and no framework enables them to easily transfer the scarce knowledge that is accessible to their individual setting. Clinicians can only take environmental concerns into consideration if the environmental impacts of respective products including their packaging are known. E.g., life cycle assessments demonstrated relevant differences in the environmental impact of inhalational versus intravenous anaesthesia. Anaesthetists are now aware of these differences, can consider this knowledge in their daily practice and opt for regional or intravenous anaesthesia when no impacts on patient outcomes are expected [14].

Importantly, most disposable products were packaged individually in some kind of plastic. In our setting, the generated plastic waste is currently disposed as solid waste. Of course, individual packaging is essential for infection prevention in many cases, but a considerable share was generated before the patient entered the OR. In our hospital, solely preoperative anaesthesia plastic waste accounts for more than 10 kg per week [15]. Preoperative plastic waste is non-infectious and could potentially be recycled in usual recycling processes [16]. Reprocessing could be facilitated if packaging did not contain mixtures of different plastics and materials. In addition, some products, such as EEG sensors, may not have to be packed in a sterile manner and their individual packaging could potentially be avoided.

In general, early enhanced recovery after surgery concepts, avoidance of adverse events and consecutive timely discharge may reduce resource consumption.

Overall, our analysis demonstrates that principles of circular economy have not yet been implemented in hospital patient care. On the one hand, industrial innovations for products and packaging are needed. On the other hand, clinicians, hygiene and sustainability experts need to work together on guidelines for sustainable infection control [17].

### Manufacturer information and literature search

Our online search on manufacturers’ information and our literature search revealed a lack of accessible data on the products’ environmental impacts. Medical societies around the globe are concerned about the climate impact of their fields’ clinical work and call for more environmentally sustainable healthcare [18–20]. To make informed decisions on which products to use in daily routine, independent LCAs according to ISO 14040/44 standards are essential [21, 22]. The development of a comprehensive life cycle inventory database, as Sherman et al. proposed, is an essential step towards transparency [17]. Currently, relevant publications either do not exist, are not available in the PubMed database or are not indexed by the keyword “LCA”, making it difficult for clinicians to quickly identify them. New formats, such as the HealthcareLCA database, may help to make relevant information more easily accessible [23]. It is noteworthy that our online search on manufacturers’ information did not identify any independent LCAs. The sustainability aspects reported for 10% of the products cannot be considered sufficient to guide decision-making. About one third of manufacturers published a general sustainability report. However, these do not contain specific information on the environmental impacts of their products. It should be discussed whether manufacturers should be held accountable for providing such information.

### Food and transportation

The majority of meals our patients consumed at the hospital contained meat or fish. Several studies have shown that plant-based meals have the potential to lower environmental impacts and reduce health risks especially in high-income countries [24, 25]. Consequently, hospitals should consider offering more plant-based alternatives. To reduce hospitals´ food waste, patients could individually choose their meal sizes.

In our setting patients travelled almost four times to the hospital for their treatment. It has been estimated that all transportation, including patient transportation, contributes 7% of the overall healthcare’s carbon footprint [2]. Digitalisation including telemedicine may reduce this impact [26, 27]. In addition, optimized scheduling of appointments, improved access to public transportation, and incentives that make bicycling more attractive may help decrease the environmental impact of transportation.

### Limitations

Our analysis comes with several limitations. First, this is a pilot project on five patients with considerable variability in their time spent in the OR and in the hospital. Data on more patients is essential to generate more precise estimations of the resources utilised. Even though clinicians usually cannot impact energy, gas and water usage, this data is crucial, because these variables have been shown to be major contributors to healthcare´s carbon footprint [2, 11].

In some cases, determining the category of a disposable versus reusable product was ambiguous, (e.g. for disinfectant (disposable)). Notably, our definition of reusable products included not only items that were reprocessed after usage, but also products that were disposed after usage for multiple patients without being reprocessed e.g., sharp containers and ventilation tubes. Another limitation is the inclusion of patients regardless of whether they had already undergone prior examinations for the upcoming surgery, which led to variations in the number of products used in the preoperative phase. Additionally, we only included the first postoperative visit at the thoracic surgery outpatient clinic and lack information on possible further visits necessary for subsequent treatments. Therefore, the resource consumption of an average thoracic patient’s treatment might be even higher. Importantly, resource consumption might be significantly higher if products utilised for procedures that were not taking place in direct approximation to the patient, such as pathology or blood testing, would have been included in our analysis. Another limitation is that we relied on interviews and our experiences regarding the quantity of products used at the ward such as for drawing blood. While the field of thoracic surgery is developing, the number of robotic-assisted-thoracoscopic surgeries will likely increase. These are more resource-intensive than VATS, underlining the need for thoracic surgery societies to contribute to future solutions [28].

## Conclusion

We found that a considerable amount of resources is utilised for the treatment of a VATS patient. The majority of products were used in the OR by anaesthesia and surgery staff. Overall, most products were disposable, and their packaging contained plastic. From a clinician’s perspective, data on the environmental impacts of products used in daily routine is scarce hampering informed purchasing choices. Information from independent LCAs concerning not only medical products but also hospital food, transportation and other areas is necessary to prioritise the most important actions to decrease the carbon footprint in healthcare. Further, interdisciplinary research is essential to leverage circular economy principles in hospital care.

## Electronic supplementary material

Below is the link to the electronic supplementary material.

Supplementary Material 1 Patient product usage across different stages of hospital treatment.

## Data Availability

The generated dataset can only be made available upon reasonable request via the corresponding author considering possible legal restrictions and after ethics approval.
